# Calvarium Subperiosteal Hematoma in a 12-Year-Old Boy

**DOI:** 10.7759/cureus.16550

**Published:** 2021-07-22

**Authors:** Yuichiro Yoneoka, Yasuhiro Seki, Katsuhiko Akiyama

**Affiliations:** 1 Neurosurgery, Uonuma Institute of Community Medicine, Niigata University Medical and Dental Hospital, Minami-Uonuma, JPN; 2 Neurosurgery, Uonuma Kikan Hospital, Uonuma Institute of Community Medicine, Niigata University Medical and Dental Hospital, Minami-Uonuma, JPN

**Keywords:** calvarium subperiosteal hematoma, cephalohematoma, head injury, juvenile, tranexamic acid, prednisolone, irrigation, computed tomography scan, computed tomography angiogram, needle aspiration

## Abstract

Calvarium subperiosteal hematoma (C-SPOH) is extremely rare in juveniles. We present an extremely rare case of juvenile C-SPOH and a review of the literature. A 12-year-old boy hit his head hard against another player’s head during a soccer game. On the next day of the game (Day 02), he noticed a soft bump on the left parietal region. On Day 04, he saw a local physician and was diagnosed with a subgaleal hematoma. The hematoma grew larger, up to twice the size of that on Day 04 and it became more painful over the next five days. A CT scan on Day 10 showed a subcutaneous hematoma that did not cross the suture lines. Aspiration using a syringe with an 18-gauge needle obtained about 45 mL liquefied hematoma and caused the bump collapse with relief of the pain. On Day 12, however, he presented the same bump with similar pains as on Day 10. CT angiography revealed no vascular anomalies or disruptions. A blood sampling test demonstrated normal blood coagulation ability without thrombocytopenia or malnutrition. A second aspiration obtained 45 mL liquefied hematoma. In the second procedure, the hematoma cavity was irrigated with normal saline solution (about 5 mL x 4). He took 250 mg tranexamic acid three times a day and 5 mg prednisolone three times a day for four days. On Day 15, his C-SPOH was not tense and not painful. On Day 22, the periosteal hematoma remained soft and shrunk. A follow-up CT scan showed the complete disappearance of the subperiosteal hematoma on Day 57. The boy has returned to soccer-playing activity without sequelae. This case suggests that 1) C-SPOH can be found in healthy juveniles; 2) Neovascularization along the wall of the C-SPOH cavity may contribute to the formation of the C-SPOH; 3) A simple aspiration of the liquefied SPOH may fail to cure it in juveniles.

## Introduction

Juvenile calvarium subperiosteal hematoma (C-SPOH) is extremely rare. A C-SPOH also known as a cephalohematoma develops during the hours or days following birth in neonates [1]. Because the fluid collection is between the periosteum and the skull, the boundaries of a cephalohematoma are defined by the underlying bone [1]. In other words, a cephalohematoma is confined to the area on top of one of the cranial bones and does not cross the midline or the suture lines [1]. Based on this definition, juvenile C-SPOH (juvenile cephalohematoma not crossing the suture lines) has not been reported yet to the best of our knowledge based on a review of the literature. In this report, we describe a juvenile case of C-SPOH and review the literature.

## Case presentation

**Figure 1:**
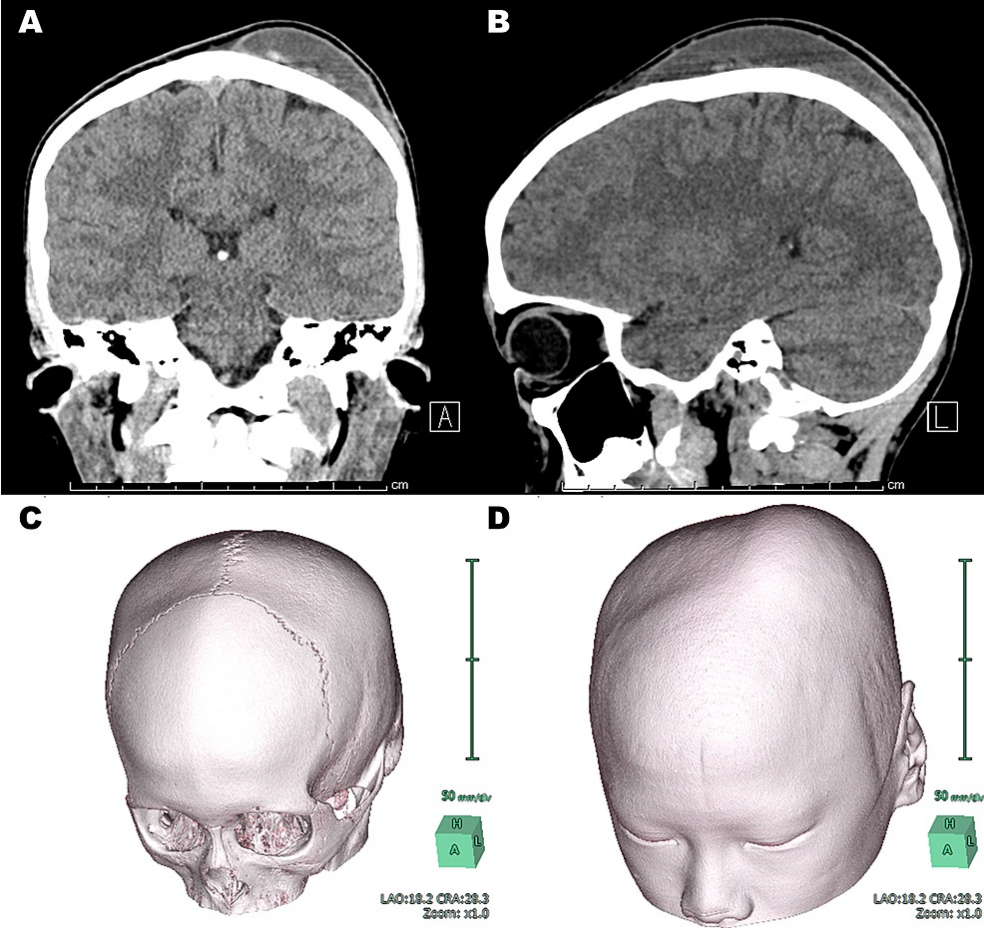
MPR images of CT scan and volume rendering. A coronal MPR image shows subcutaneous hematoma (A) as does a sagittal MPR image (B). The hematoma does not cross the suture lines (C), indicating that this subcutaneous hematoma (A, B, D) is C-SPOH. MPR, multiplanar reconstruction; C-SPOH, calvarium subperiosteal hematoma

A 12-year-old boy hit his head hard against another player’s head during a soccer game. He continued to play the game despite the head injury. On the next day of the game (Day 02), the 12-year-old boy noticed a soft bump on the left parietal region. On Day 04, he saw a local physician and was diagnosed with a subgaleal hematoma. At that time, conservative therapy was selected because no other abnormalities were found upon physical examinations. The hematoma became larger up to twice the size of that on Day 04 and more painful over the next five days. He visited our outpatient clinic on Day 10. The tense bump was localized in the left parietal region and was not pulsatile. A CT scan showed a subcutaneous hematoma (Figure 1A,B) that did not cross the suture lines (Figure 1C,D).

**Figure 2:**
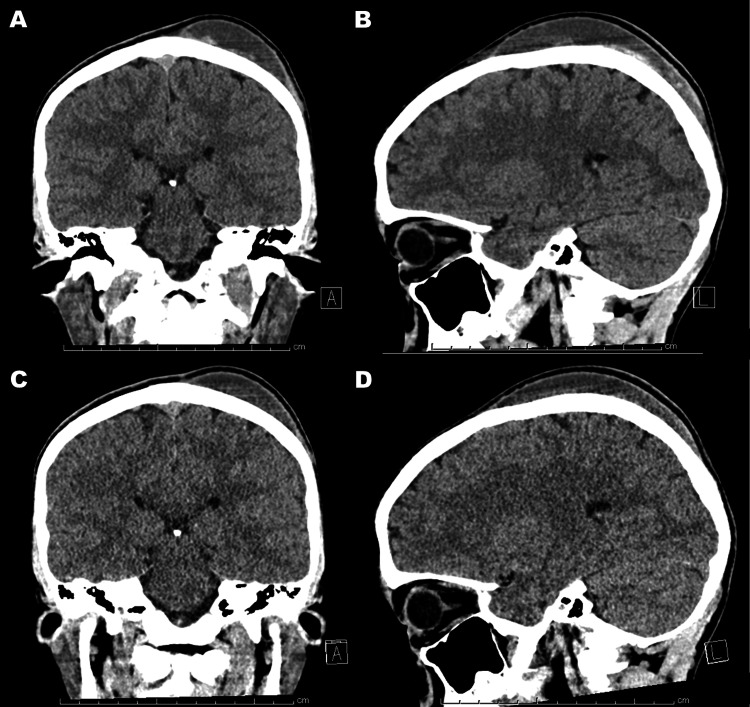
CT scan images before a simple needle aspiration and after five days. A simple needle aspiration of the C-SPOH (A, B) fails to cure, and accumulation is allowed again (C, D). C-SPOH, calvarium subperiosteal hematoma

Aspiration using a syringe with an 18-gauge needle obtained about 45 mL liquefied hematoma and made the bump collapse with relief of the pain. On Day 12, however, he visited our outpatient clinic complaining of a bump similar to the previous one (Figure 2A,B) with pains similar to those experienced on Day 10 (Figure 2C,D).

**Figure 3:**
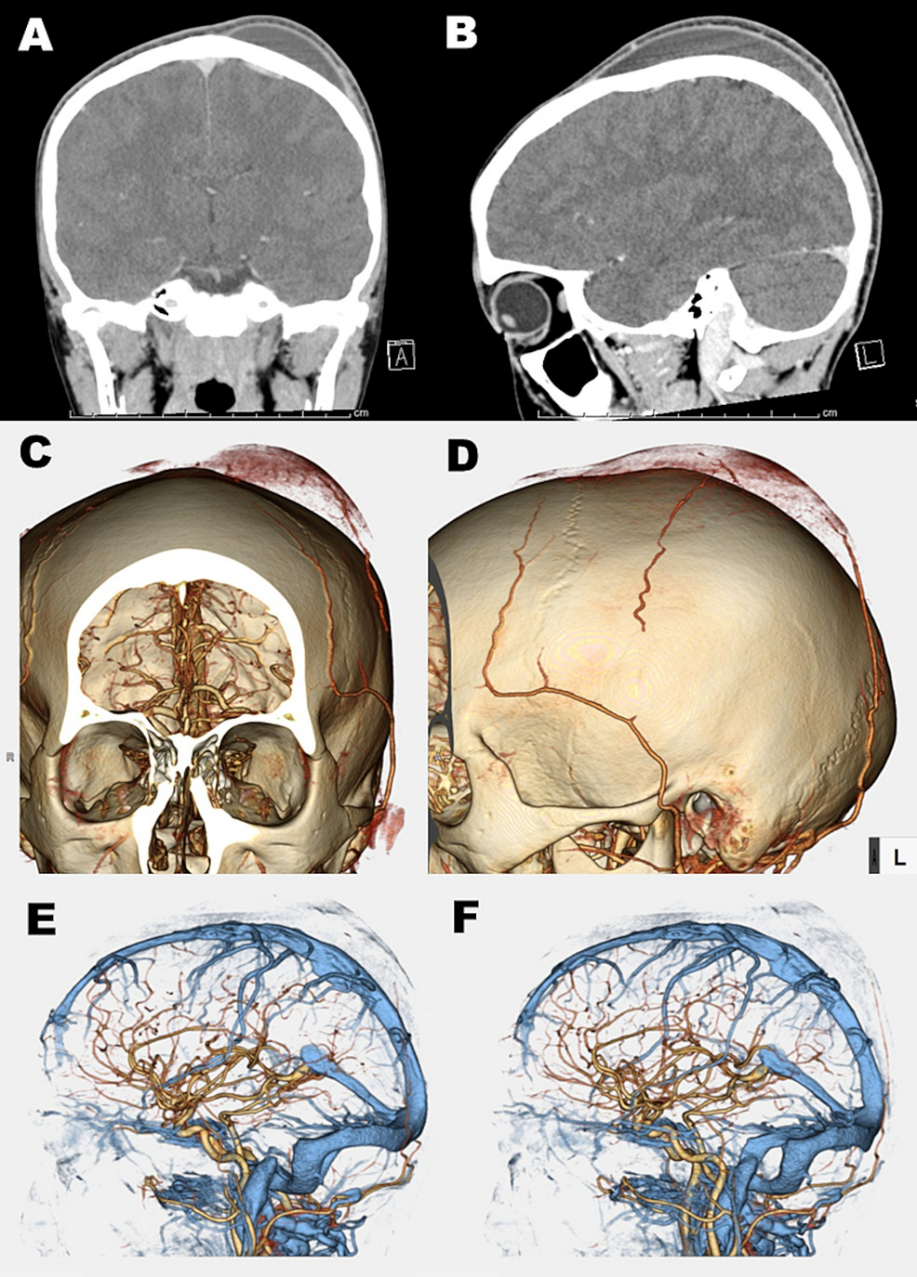
Postcontrast CT scan images and three-dimensional CT angiography. Postcontrast CT scan images show enhancement along the subperiosteal hematoma cavity (A, B), which is confirmed by three-dimensional CT angiography (C, D). Damage of the superior sagittal sinus or adjacent veins was not demonstrated (E, F).

Physical examinations revealed no abnormal findings except for the left parietal bump. A careful interview failed to detect other traumatic histories including domestic violence. A follow-up CT scan showed reaccumulation of the C-SPOH not crossing the suture lines (Figure 2C,D). CT angiography revealed no vascular anomalies, no vascular disruptions, but a partially enhanced wall of the subperiosteal hematoma cavity (Figure 3A-D).

**Figure 4:**
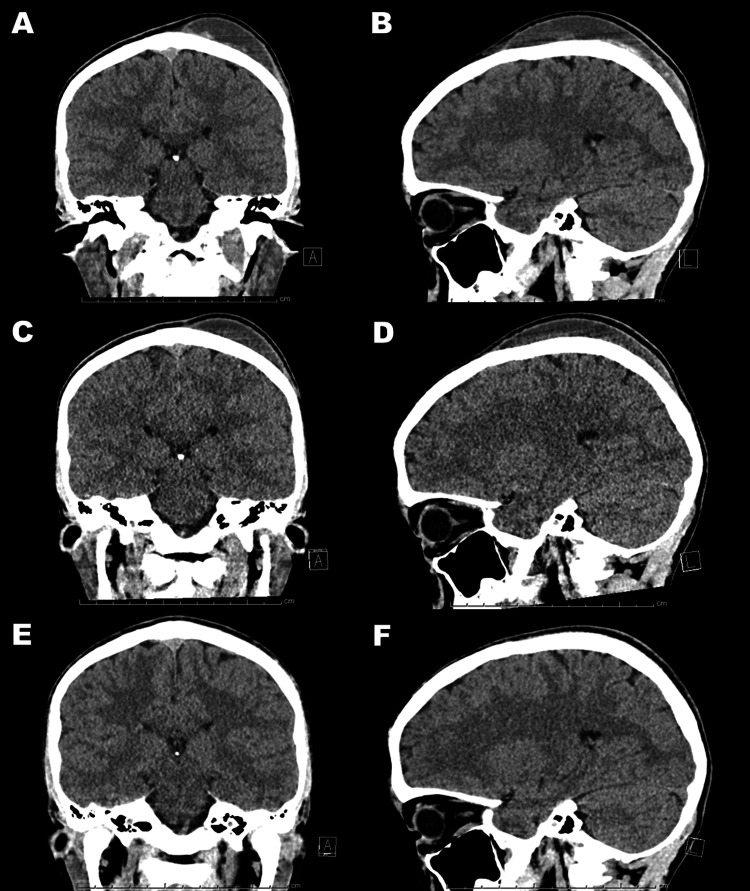
Follow-up CT scan images. The C-SPOH (A, B), through the reaccumulating (C, D), shrinks to provide the cure, which is demonstrated on follow-up CT scan of Day 57 (E, F). C-SPOH, calvarium subperiosteal hematoma

A blood sampling test demonstrated normal blood coagulation ability without thrombocytopenia or malnutrition. A second aspiration obtained 45 mL liquefied hematoma. In the second procedure, the hematoma cavity was irrigated with normal saline solution (about 5 mL x 4). He took 250 mg tranexamic acid three times a day and 5 mg prednisolone three times a day for four days. On Day 15, his subperiosteal hematoma was not tense and not painful. Peroral medication of tranexamic acid and prednisolone was discontinued on Day 16. On Day 22, the periosteal hematoma remained soft and shrunk through the re-accumulation (Figure 4A-D).

A follow-up CT scan showed the complete disappearance of the subperiosteal hematoma on Day 57 (Figure 4E,F). The boy returned to soccer-playing activity without sequelae.

## Discussion

Calvarium subperiosteal hematoma is extremely rare in juveniles. To the best of our knowledge, only one case is known [2]. Higashi and his colleagues reported a huge recurrent subperiosteal hematoma in a nine-year-old boy [2]. They described subperiosteal hematoma in the figures of their report [2] which obviously crossed the suture lines and initiated from the periorbital region. Thus, their subperiosteal hematoma should be differentiated from our case of C-SPOH.

Cephalohematoma is a subperiosteal collection of blood caused by rupture of vessels beneath the periosteum (usually over the parietal or occipital bone), which presents as swelling that does not cross suture lines [3]. The majority of cephalohematomas resolve spontaneously over the course of a few weeks without any intervention [3]. In general, however, cephalohematoma is known as one of the neonatal birth injuries, and juvenile cephalohematoma has not been reported, even in a young population with hematological disorders. In our case, hematological disorders neither have been detected nor have an unusual head injury or violation to the head except for the heading in the soccer game. The pathogenesis of this C-SPOH (cephalohematoma) still remains unclear. Postcontrast three-dimensional CT angiography showed a partially enhanced wall of the subperiosteal hematoma cavity (Figure 3A-D). Neovascularization along the wall of the subperiosteal hematoma cavity (Figure 3C-D) depicted by enhanced CT scan may contribute to the formation of a subperiosteal hematoma. Hashimoto et al. reported on a ‘cotton wool-like staining’ seen on their angiographic evaluations of chronic subdural hematomas (CSDHs), mimicking the abnormal vasculature of the membranous layers [4]. Entezami et al. reported that this capillary pattern is encountered during angiograms [5] and supports the apparent neovascular proliferation seen in the outer membrane of these hematomas [4-5]. Pathogenesis of formation of our subperitoneal hematoma could be similar to that of CSDH. The review by Edlmann et al. focuses on several key processes involved in CSDH development: angiogenesis, fibrinolysis and inflammation [6], which will help our understanding and investigation of the pathogenesis of C-SPOH. Although a simple aspiration could relieve the pains caused by the parietal cephalohematoma in our case, prompt recurrence of the cephalohematoma occurred. After the second aspiration, we gave the patient tranexamic acid and prednisolone. The conservative management of CSDH with tranexamic acid is both a safe and effective alternative in the absence of life-threatening symptoms [7]. From the viewpoint of steroid application to CSDH, current evidence implicates a potentially beneficial role of dexamethasone in the management of CSDH [8]. Pharmacological agents are a particular focus of CSDH management currently, and a wealth of studies on steroids will hopefully lead to more harmonized, evidence-based practice regarding this in the near future [9]. Although the therapeutic evidence of CSDH cannot be adapted to cephalohematoma, we used them as reference. It is difficult to assess whether our application of prednisolone to the cephalohematoma is effective or not as well as irrigation of the cephalohematoma cavity. In fact, a simple single aspiration failed to cure the cephalohematoma, so some additional intervention would be necessary. The key processes involved in CSDH development: angiogenesis, fibrinolysis, and inflammation [6] possibly also contributed to the formation of C-SPOH. We applied irrigation, tranexamic acid, and steroids in our case. After their application, in fact, the C-SPOH settled and shrunk smoothly. Over time, the bulge may feel harder as the collected blood calcifies. If that hematoma had not been treated, albeit rarely, large calcified cephalohematomas would need surgical treatment with a certain probability [10-11]. Thus, appropriate treatment methods are desired. One example is not sufficient to discuss the pathogenesis and the treatment, nonetheless, it will give us some kind of clue for C-SPOH. As CT use has increased rapidly, radiation protection is important, particularly among children [12]. Nevertheless, although clinical benefits should outweigh the small absolute risks, radiation doses from CT scans ought to be kept as low as possible and alternative procedures, which do not involve ionizing radiation, should be considered if appropriate [13].

## Conclusions

This case suggests that 1) C-SPOH can be found in healthy juveniles; 2) Neovascularization along the wall of the C-SPOH cavity may contribute to the formation of the C-SPOH; 3) A simple aspiration of the liquefied SPOH may fail to cure it in juveniles. Optimal therapeutic intervention for juvenile C-SPOH (cephalohematoma) remains unclear as well as its pathogenesis. Further accumulation of cases of this extremely rare juvenile cephalohematoma is warranted.
